# A Comprehensive Analysis of Surgical Outcomes for Spinal Fractures in Patients With Ankylosing Spondylitis: A 13-Year Prospective Study

**DOI:** 10.7759/cureus.76036

**Published:** 2024-12-19

**Authors:** Md. Shah Alam, Md. Ziaul Hasan, Abdullah Al Mamun Choudhury, Md. Sarwar Jahan, OZM Dastagir, Mohammad R Amin Molla, Mohammed A Islam

**Affiliations:** 1 Spine Surgery, Bangladesh Spine & Orthopaedic Hospital, Dhaka, BGD; 2 Orthopaedic and Spine Surgery, National Institute of Traumatology and Orthopaedic Rehabilitation (NITOR), Dhaka, BGD; 3 Spine Surgery, National Institute of Traumatology and Orthopaedic Rehabilitation (NITOR), Dhaka, BGD; 4 Orthopaedic, Spine, and Trauma Surgery, National Institute of Traumatology and Orthopaedic Rehabilitation (NITOR), Dhaka, BGD

**Keywords:** ankylosing spondylitis, as, mri in spinal trauma, spinal cord injury, spinal injury

## Abstract

Introduction: Ankylosing spondylitis (AS), a chronic inflammatory spondyloarthropathy affecting the spine, progressively leads to increased spinal stiffness. This condition increases the risk of spine fractures in patients, even from trivial injuries. The process of slow bone formation within the ligaments of the spine and the fusion of the spinal diarthrosis contribute to the most prominent symptom of progressive stiffness of joints, predominantly affecting the spine and sacroiliac joints. In advanced AS, the ossification of the paraspinal tissues and inflammatory osteitis result in a spine that is brittle and fragile, making it susceptible to fractures.

Objective: This study aimed to observe and analyze the postoperative surgical outcomes after spine fracture fixations in patients with AS, focusing on the intricacies that exist in diagnosis and surgical treatment.

Methods: A prospective interventional study was conducted in a tertiary care center during the period from 2010 to 2023. This study evaluated 25 cases of spinal fractures diagnosed with AS.

Results: The mean patient age was 58.61 ± 18.54 years, with the majority being male (72%; n = 18) over female (28%; n = 7). The predominant cause of injury was falling from a height (56%, n = 14), and the most frequent fracture location was the C7-T1 (n = 10). Fracture distribution included cervical (32%, n = 8), lumbar (28%, n = 7), thoracic (24%, n = 6), and thoracolumbar (16%, n = 4). Sixteen percent (n = 4) of the patients had no fracture on X-rays but eventually were diagnosed with a fracture on an MRI. Following surgery, there was a notable improvement in neck disability index (NDI) and visual analog scale (VAS) scores (p < 0.05). Neurological status improvement was observed, with 24% (n = 6) of patients improving to Frankel D and 68% (n = 17) to Frankel E. The postoperative complication noted was wound infection in 8% (n = 2) of the cases.

Conclusion: This study emphasizes the effectiveness of anterior surgical interventions for treating spine fractures in AS patients, as shown by significant improvements in both quality of life and neurological status. Despite the complexities of treating spinal fractures in this population, marked by a high incidence of cervical and lumbar injuries, our results demonstrate positive outcomes. The occurrence of postoperative complications such as sepsis and instrumentation failure necessitates careful surgical planning and attentive postoperative care. This study offers valuable insights for enhancing treatment strategies and patient management in cases of spine trauma involving AS.

## Introduction

Ankylosing spondylitis (AS), a chronic inflammatory spondyloarthropathy affecting the spine, progressively leads to increased spinal stiffness. This condition increases the risk of spine fractures in patients, even from trivial injuries. The ossification of the paraspinal tissues and inflammatory osteitis result in a spine that is brittle and fragile, making it susceptible to fractures [1,2]. It has a prevalence of 0.1% to 1.4%, primarily affects men, with a male-to-female ratio of approximately 3-4:1, and manifests between the ages of 15 and 35 [3,4]. The process of slow bone formation within the ligaments of the spine and the fusion of spinal diarthrosis contribute to the most prominent symptom of progressive stiffness of joints, predominantly affecting the spine and sacroiliac joints.

The extensive inflammation causes structural alterations and the production of syndesmophytes, which fuse spinal segments [5]. The immobility of ankylosed vertebrae, along with the disease's poor bone mineral density, makes them vulnerable to fractures [6]. The lower cervical spine and the dorso-lumbar spine are the most commonly documented vertebral fractures in AS patients [7,8]. Fractures in the cervical spine of AS patients are an extremely fragile condition that frequently results in further neurological impairments [1,2].

While conservative management for AS has been associated with a higher mortality rate [1], surgical intervention involving instrumentation, reduction of fracture, and decompression is now the preferred approach. The management of cervical AS remains a subject of debate, particularly regarding the most effective surgical techniques. Various surgical methods have been proposed, including the anterior procedure using a cervical plate, the posterior approach utilizing lateral mass fixation, the wiring of the interspinous region with autologous grafts, cervical pedicle screw fixation, or hybrid techniques combining anterior and posterior techniques [1,9-11].

AS renders the spine less flexible, impairing its ability to bear the same standard weights as a normal spine would. Research has shown that a decrease in bone mineral density (BMD) is an early occurrence in the progression of AS, caused by inflammation, resulting in enhanced bone resorption [12]. The kyphotic deformation often seen in these cases further predisposes the ankylosed and osteoporotic spine to stress fractures under relatively low forces and loads [4]. Advanced AS causes a fused, fragile spine due to pervasive paraspinal ossification and inflammatory osteitis [4,12,13].

Individuals with AS can sustain fractures even with minimal or no known cause of trauma. Fractures in AS patients, particularly those occurring at the cervico-thoracic junction of the spine, should be treated as high-risk injuries, given their potentially severe implications.

Shearing fractures are the most unstable [14]. Spinal fractures in AS patients can lead to severe neurological compromise or cause complications such as hemothorax or rupture of the aorta [14]. Furthermore, there is a risk of further neurological impairment due to the displacement of the fractured segment, often seen in hyperextension injuries [14].

This study aimed to observe and analyze the postoperative surgical outcomes after spine fracture fixation in patients with AS.

## Materials and methods

This prospective interventional study was conducted at a tertiary care center from June 2010 to March 2023. This study included 25 (100%) cases of AS, selected according to specific inclusion and exclusion criteria.

Table 1 presents the eligibility criteria for participation in the study. Patients aged 31-70 with AS and spinal fractures involving three columns were included. Those with uncontrolled diabetes, previous surgeries, or acute illnesses such as renal or pancreatic diseases and heart issues were excluded from the study. These criteria help us focus on a specific group for accurate results.

**Table 1:** Inclusion and exclusion criteria for the AS spinal fracture study DM: diabetes mellitus; AS: ankylosing spondylitis

Inclusion Criteria	Exclusion Criteria
Patients aged between 31 and 70 years	Patients with uncontrolled DM
Patients with ankylosing spondylitis	Patients with prior surgical history
Patients with spinal fractures	Patients with any history of acute illness
Fractures involving three columns of the spine	Patients with any history of acute illness (renal or pancreatic diseases, ischemic heart disease, etc.)

Study methodology

The epidemiological data were noted in the patient's medical record. Surgical intervention was considered when the integrity of the spine was compromised by the fracture, if there was a neurological deficit at diagnosis, during hospital stay, or in cases where both of these factors were present. The spine fractures were classified using the AO system. The injury level and neurological status of each patient were ascertained through clinical evaluation and radiological imaging. To assess neurological conditions, we employed Frankel's neurological classification system.

Upon admission, radiographs were taken (anteroposterior, lateral, and oblique views). If initial X-ray imaging tests did not confirm a fracture, but clinical suspicion persisted, further investigations were done using computed tomography (CT) and magnetic resonance imaging (MRI). Data regarding the duration of the surgical procedure and any intraoperative complications were meticulously documented. Each patient underwent assessment and evaluation upon admission and postoperatively until discharge from the hospital. Patients were also evaluated at three, six, nine, and 12 months after the operation and thereafter annually. The visual analog scale (VAS), neck disability index (NDI), and Frankel grading were all assessed at each follow-up. X-rays and CT scans were used to determine implant position and fracture segment fusion. The study was approved by the ethical review committee of the institution.

Statistical analysis

Data were methodically recorded using a predefined data collection form. Statistical analyses were conducted using IBM SPSS Statistics for Windows, Version 20 (Released 2011; IBM Corp., Armonk, New York). A p-value < 0.05 was considered significant.

## Results

A significant portion of the participants fall within the age range of 51-60 years, making up 40% (n = 10) of the total. Participants aged 41-50 constitute 28% (n = 7) of the study population, while those over 60 account for 20% (n = 5). The mean age of the participants is 58.61 years, with a standard deviation of 18.54, indicating a wide age range within the study group (Table 2).

**Table 2:** Age distribution of the study participants The table presents the number of patients (N) in each age group and the corresponding percentage distribution (P%). It also demonstrates the mean age and standard deviation to show the study group's age range and central tendency.

Age Group	N	P (%)
31-40 years	3	12
41-50 years	7	28
51-60 years	10	40
>60 years	5	20
Mean age (years)	-	58.61 ± 18.54

Table 3 presents a detailed breakdown of the gender distribution among the study participants. The majority of the participants were male, comprising 72% (n = 18) of the total study population. In comparison, female participants constituted 28% (n = 7) of the population. This significant disparity in gender distribution highlights a predominance of male participants in the study.

**Table 3:** Comprehensive analysis of gender distribution among the study participants This table illustrates the gender distribution of the study participants, indicating the number (N) and percentage (P) of male and female participants.

Gender	N	P (%)
Male	18	72
Female	7	28

The majority of injuries were due to falls from height, comprising 56% (n = 14) of the total. Road traffic accidents were responsible for 44% (n = 11) of injuries. This distribution highlights the predominant causes of injuries in the study group (Table 4).

**Table 4:** Mechanisms of injury among the study participants The table presents a breakdown of the mechanisms of injury, the number of patients (N), and the corresponding percentages (P%). The data reveal the main factors that led to injuries among the participants in the study.

Mechanism of Injury	N	P (%)
Fall from height	14	56
Road traffic accident	11	44

The majority of fractures occurred at the C7/T1 level, comprising 40% (n = 10) of the total. Fractures at the C6/C7 level accounted for 32% (n = 8). This distribution highlights the most common levels of fractures in the study group (Table 5).

**Table 5:** Distribution of fracture levels The table presents a breakdown of the fracture levels, the number of patients (N), and the corresponding percentages (P%). The data indicate the frequency and distribution of different fracture levels within the study group.

Level of Fracture	N	P (%)
C3/C4	2	8
C4/C5	2	8
C5/C6	3	12
C6/C7	8	32
C7/T1	10	40

The clinical characteristics highlight that the average height of participants is 163.5 cm, with a weight of 67.06 kg and a body mass index (BMI) of 27.50 kg/m^2^. The mean operative time was 4.20 hours, the mean blood loss was 152.74 mL, and the average hospital stay was 24.20 days (Table 6).

**Table 6:** Clinical characteristics of the study participants The table presents the mean values and standard deviations (SD) for various clinical characteristics of the study group. These metrics include height, weight, body mass index (BMI), mean operative time, mean blood loss, and mean hospital stay.

Clinical Characteristics	Mean ± SD
Height (cm)	163.5 ± 7.9
Weight (kg)	67.06 ± 8.92
Body mass index (kg/m^2^)	27.50 ± 6.8
Mean operative time (hours)	4.20 ± 1.21
Mean blood loss (mL)	152.74 ± 84.27
Mean hospital stay (days)	24.20 ± 19.21

The functional outcomes and neurological status of the patients were assessed preoperatively and postoperatively. The average preoperative NDI score was 17.5 ± 8.9, which improved significantly to 10.7 ± 5.8 at the final follow-up. Similarly, the mean VAS score for pain decreased from 6.1 ± 3.4 preoperatively to 3.1 ± 1.7 postoperatively, indicating significant improvement (p < 0.05) (Table 7).

**Table 7:** Functional outcome of the study participants The table presents the preoperative and postoperative mean values and standard deviations (SD) for the neck disability index (NDI) and visual analog scale (VAS), along with the corresponding p-values indicating statistical significance.

Functional Outcome	Preoperative	Postoperative	P-value
Neck disability index (NDI)	17.5 ± 8.9	10.7 ± 5.8	<0.05
Visual analog scale (VAS)	6.1 ± 3.4	3.1 ± 1.7	<0.05

The results indicate significant neurological improvement post-surgery. Before surgery, no patients were in the Frankel E category, which denotes normal motor and sensory function. After the surgery, 17 individuals were categorized as Frankel E. Additionally, patients in the Frankel B and C categories improved to higher functional levels, indicating the effectiveness of the surgical intervention (Table 8).

**Table 8:** Changes in the neurological status of the study participants The neurological status of patients was assessed using the Frankel classification preoperatively and postoperatively. The table shows the number of patients in each Frankel category before and after surgery.

Neurological Status	Preoperative	Postoperative
Frankel A	2	2
Frankel B	6	0
Frankel C	10	0
Frankel D	7	6
Frankel E	0	17

The majority of patients (72%, n = 18) experienced no complications. Nerve root injury and miscellaneous complications each affected 8% (n = 2) of the patients. Sepsis, instrumentation failure, and wound infection occurred in 4% (n = 1) of patients. This distribution provides insight into the safety and risks associated with the procedures in the study (Table 9).

**Table 9:** Analysis of complications among the study participants The table presents the number of patients (N) who experienced each type of complication and the corresponding percentage distribution (%). This analysis highlights the incidence and types of complications encountered in the study.

Complications	Number of Patients	Percentage Distribution (%)
No complications	18	72
Nerve root injury	2	8
Sepsis	1	4
Instrumentation failure	1	4
Wound infection	1	4
Miscellaneous complications	2	8

**Figure 1:**
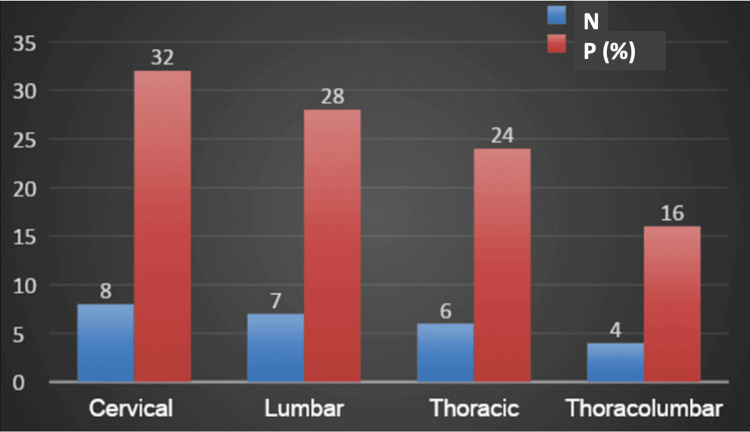
Distribution of fracture locations among the study participants The bar chart illustrates the distribution of fractures in different regions of the spine among the study participants. The blue bars represent the number of patients (N) with fractures in each region, while the red bars indicate the corresponding percentage distribution (P%).

Figure 1 shows that the cervical region had the highest percentage of fractures (32%, n = 8), followed by the lumbar region (28%, n = 7), the thoracic region (24%, n = 6), and the thoracolumbar region (16%, n = 4). This distribution highlights the prevalence of fractures in different regions of the spine among the study participants.

## Discussion

Spinal injuries in AS patients exhibit unique characteristics. The spine in AS patients becomes rigid and similar to a long bone, characterized by osteoporosis and cervical kyphosis. Consequently, due to these factors, most AS patients sustain injuries from low-energy injuries or trivial falls [11].

The debate over operative versus non-operative management for vertebral fractures in AS patients is well documented, yet the variations within surgical methods or approaches are less explored. Spinal fractures in AS patients tend to result in higher rates of neurological complications compared to those with non-ankylosed spinal fractures. Options for non-surgical management include bed rest, the use of roto-rest beds, and immobilization using braces or halo vests [7]. Surgical interventions include fixation by posterior or anterior or a combination of both. Although treatment guidelines have evolved over time, both earlier and more recent studies agree that surgery is advisable in cases of progressive neurological impairments. The trend toward minimally invasive surgical techniques, sometimes in conjunction with decompression, is also becoming increasingly prevalent [15]. Cervical fractures occur more commonly than thoracolumbar fractures in AS patients [15].

In this study, a prospective analysis of outcomes of spinal fusion surgery in AS patients with spinal fractures was conducted. The mean age was 58.61 ± 18.54 years. In a study by Luksanapruksa et al., the mean age was 68.61±11.61 years in their posterior approach group [11]. The study by Sapkas et al. reported a mean age of 55 years (range 38-80) and 56.5 years (range 23-69) for males and females, respectively [14]. Studies by Kurucan et al. [15] and Longo et al. [16] had mean ages of 67.4 ± 13.5 years and 59.2 years, respectively.

In our study, the majority of the patients were male (72%, n = 18). Luksanapruksa et al., Sapkas et al., Kurucan et al., and Longo et al. also found male predominance in their studies [11,14-16]. The most common injury mechanism was fall from height, which was similar to the results of Sapkas et al. [14].

In our research, the most commonly observed injury level was C7-T1, with C6-C7 being the second most common. We compared this with the study by Luksanapruksa et al., who noted C6-7, C4-5, and C5-6 as predominant injury levels [11]. Additionally, Sapkas et al. observed that fractures in the cervical spine typically were compression injuries, predominantly at the C6 and C7 vertebrae, followed by the C2 vertebrae [14]. In alignment with this, Longo et al.'s research also highlighted C6-7, C5-6, and C4-5 as the principal levels of injury [16].

In patients with AS, cervical fractures are more commonly reported than thoracolumbar fractures. In our study, the majority of patients presented with cervical fractures, followed by lumbar fractures. A study by Westerveld et al. reported that 77.5% (n = 267) of fractures occurred in the subaxial cervical spine [7]. In a recent analysis of the National Inpatient Sample (NIS) database from 2005 to 2011, Lukasiewicz et al. found that cervical spine fractures constituted 53% (n = 498) of cases, thoracic spine fractures 41.9% (n = 393), lumbar spine fractures 18.2% (n = 171), and sacral fractures 1.5% (n = 14) [17]. Similarly, Kurucan et al. reported a similar distribution, with cervical spine fractures at 47.4% (n = 456), thoracic at 37.5% (n = 360), lumbar at 13.7% (n = 132), and sacral fractures at 1.4% (n = 13) [15].

In our study, the mean preoperative NDI score was 17.5 ± 8.9, which showed improvement to 10.7 ± 5.8 at the final follow-up. Similarly, the mean preoperative VAS score of 6.1 ± 3.4 improved to 3.1 ± 1.7 at the final assessment. In the current study, preoperatively, 8% (n = 2) of the patients had Frankel A, 24% (n = 6) had Frankel B, 40% (n = 10) had Frankel C, and 28% (n = 7) had Frankel D neurology. Postoperatively, 8% (n = 2) of the patients remained in Frankel A, 24% (n = 6) improved to Frankel D, and 68% (n = 17) improved to Frankel E neurology.

According to Sapkas et al., preoperatively, 15% (n = 3) were categorized as Frankel A, 10% (n = 2) as Frankel B, 20% (n = 4) as Frankel C, 5% (n = 1) as Frankel D, and 50% (n = 10) as Frankel E. Postoperatively, 15% (n = 3) remained Frankel A, 20% (n = 4) became Frankel D, and 65% (n = 13) became Frankel E [14]. According to the study by Longo et al., the majority of patients experienced neurological improvements of at least one Frankel grade after surgery; this was true for 78% (n = 31) of the patients in the combined procedure, 79% (n = 11) in the anterior procedure, and 70% (n = 7) in the posterior procedure [16].

In our present study, we found wound infection in 8% (n = 2) of the cases. Longo et al. reported pneumonia, wound infection, and deformity as the most common complications following surgery [16]. Teunissen et al. found pneumonia, respiratory failure, altered mental status, UTI, and wound infection [18]. Rustagi et al. found pneumonia and respiratory failure to be postoperative complications in their study [19]. Lukasiewicz et al. found UTI, acute kidney injury (AKI), and pneumonia in their study [17]. Altun et al. found pseudoarthrosis, wound infection, and pneumonia in their study [20].

Study limitations

The limitations of our study include a small sample size. Additionally, conducting a multicenter study would better represent the real-world scenario of spinal injuries in AS patients, providing more generalizable and comprehensive data across different healthcare settings.

## Conclusions

In our study, most patients had no complications after undergoing anterior surgery. Their functional outcome improved as assessed by the NDI and VAS. Their neurological status also improved after the surgical stabilization. Surgical treatment for spinal injuries in AS patients is typically effective and beneficial, usually resulting in improved neurological function unless complete paraplegia is pre-established.

## References

[1] Altenbernd J, Bitu S, Lemburg S (2009). Vertebral fractures in patients with ankylosing spondylitis: a retrospective analysis of 66 patients. Rofo.

[2] Caron T, Bransford R, Nguyen Q, Agel J, Chapman J, Bellabarba C (2010). Spine fractures in patients with ankylosing spinal disorders. Spine.

[3] Bechterew VM (1979). The classic stiffening of the spine in flexion, a special form of disease. Clin Orthop Relat Res.

[4] Linden S van der, Heijde D van der, Braun J (2006). Ankylosing spondylitis. Kelley's Textbook of Rheumatology.

[5] Wanders A, Landewe R, Dougados M (2005). Association between radiographic damage of the spine and spinal mobility for individual patients with ankylosing spondylitis: can assessment of spinal mobility be a proxy for radiographic evaluation?. Ann Rheum Dis.

[6] Davey-Ranasinghe N, Deodhar A (2013). Osteoporosis and vertebral fractures in ankylosing spondylitis. Curr Opin Rheumatol.

[7] Westerveld LA, Verlaan JJ, Oner FC (2009). Spinal fractures in patients with ankylosing spinal disorders: a systematic review of the literature on treatment, neurological status and complications. Eur Spine J.

[8] Olerud C, Frost A, Bring J (1996). Spinal fractures in patients with ankylosing spondylitis. Eur Spine J.

[9] Heineck J, Bergert H, Muller M (2007). Ventral fusion of a fracture of the cervical spine in ankylosing spondylitis and struma permagna. Unfallchirurg.

[10] Ding S, Zheng K (2010). Artificial total hip arthroplasty with collum femoris preserving for treating hip joint (Article in Chinese). Zhongguo Xiu Fu Chong Jian Wai Ke Za Zhi.

[11] Luksanapruksa P, Millhouse PW, Carlson V, Ariyawatkul T, Heller J, Kepler CK (2019). Comparison of surgical outcomes of the posterior and combined approaches for repair of cervical fractures in ankylosing spondylitis. Asian Spine J.

[12] Horst-Bruinsma IE van der (2006). Clinical aspects of ankylosing spondylitis. Ankylosing Spondylitis: Diagnosis and Management.

[13] De Peretti F, Sane JC, Dran G, Razafindratsiva C, Argenson C (2004). Ankylosed spine fractures with spondylitis or diffuse idiopathic skeletal hyperostosis: diagnosis and complications (Article in French). Rev Chir Orthop Reparatrice Appar Mot.

[14] Sapkas G, Kateros K, Papadakis SA, Galanakos S, Brilakis E, Machairas G, Katonis P (2009). Surgical outcome after spinal fractures in patients with ankylosing spondylitis. BMC Musculoskel Disord.

[15] Kurucan E, Bernstein DN, Mesfin A (2018). Surgical management of spinal fractures in ankylosing spondylitis. J Spine Surg.

[16] Longo UG, Loppini M, Petrillo S (2015). Management of cervical fractures in ankylosing spondylitis: anterior, posterior or combined approach?. Br Med Bull.

[17] Lukasiewicz AM, Bohl DD, Varthi AG (2016). Spinal fracture in patients with ankylosing spondylitis: cohort definition, distribution of injuries, and hospital outcomes. Spine.

[18] Teunissen FR, Verbeek BM, Cha TD (2017). Spinal cord injury after traumatic spine fracture in patients with ankylosing spinal disorders. J Neurosurg Spine.

[19] Rustagi T, Drazin D, Oner C (2017). Fractures in spinal ankylosing disorders: a narrative review of disease and injury types, treatment techniques, and outcomes. J Orthop Trauma.

[20] Altun I, Yuksel KZ (2016). Ankylosing spondylitis: patterns of spinal injury and treatment outcomes. Asian Spine J.

